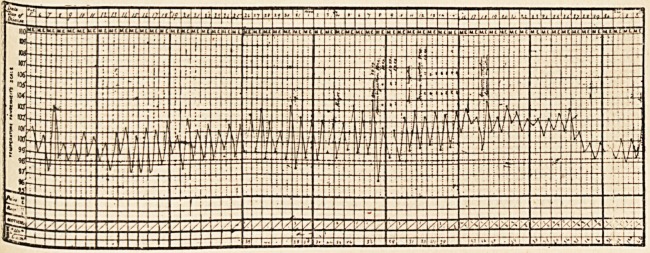# A Case of Acute Miliary Tuberculosis

**Published:** 1897-06

**Authors:** J. Michell Clarke

**Affiliations:** Physician to the Bristol General Hospital, and Professor of Pathology, University College, Bristol


### A CASE OF ACUTE MILIARY TUBERCULOSIS

### TREATED WITH ANTISTREPTOCOCCIC SERUM ON ACCOUNT OF THE PRESENCE IN THE BLOOD OF THE STREPTOCOCCUS PYOGENES ALBUS.[1]

BY

J. MICHELL CLARKE, M.A., M.D. Cantab., F.R.C.P.,

*Physician to the Bristol General Hospital, and*
*Professor of Pathology, University College, Bristol.*

---

THE points of interest in this case lie not only in the presence of S. pyogenes albus in the blood of a patient with acute miliary tuberculosis, but also in the clinical resemblance to ulcerative endocarditis.

G. J., aged 20, a labourer, was admitted on October 5th, 1896, complaining of pains in the abdomen, chiefly on the right side in the iliac region, of pains in the back, and also occasionally of pains in the penis when making water. He had also a slight cough. The pains prevented him from working; he attended as an out-patient for a short time, and as he was found to have some fever, and the pains did not improve under treatment, he was admitted.

The family history was good, and there was no evidence of tuberculosis. In March, 1896, he contracted enteric fever, and was laid up for two months; otherwise he had always been perfectly healthy.

On admission, he was well nourished: slightly dusky in aspect. He appeared weak; his temperature was 101°; pulse-rate, 112; respiration-rate, 28; tougue somewhat furred; bowels constipated. The chest was well formed and expanded well. There was a little deficient resonance, harshness of breath-sounds, and increased vocal resonance at the right apex: and over the left lung in front a few moist sounds were occasionally heard. The heart was not enlarged, and there was no murmur at this time: the sounds were loud. The area of splenic dulness was somewhat increased in size. The urine contained no sugar or albumen: it was acid: sp. gr., 1015. There was a chronic ulcer on the outer side of the right heel.

From October 5th–24th the temperature followed a marked hectic type, ranging from normal or below normal in the morning, to 102°–103° in the evening: the patient was dull, weak, listless, apathetic, and slept badly at night; his appetite was bad; the bowels were constipated, he complained of abdominal pain after they were moved; the pulse varied from 100-120; no fresh physical signs were detected in the lungs on examination. At the latter date the left ventricle of the heart was found to be somewhat dilated, and a loud blowing systolic murmur was audible over the pulmonary area. In the abdomen there was tenderness to the right of the umbilicus, with some rigidity of the abdominal

[1] Read at the April Meeting of the Bristol Medico-Chirurgical Society.

wall here, and a feeling of matting together of the abdominal contents. The patient was obviously losing flesh.

During the night of October 30th he had a series of rigors, in which the temperature rose to 103.5°. Next day, October 31st, he was very weak: pulse was 124, soft and compressible; respiration 44, shallow. The splenic dulness showed a further increase, and he complained of pain in that region. The urine contained no sugar or albumen, and it may be here mentioned that throughout the illness it gave no reaction to Ehrlich's test. There was no optic neuritis; there were no tubercles in the choroid; no retinal hemorrhages. Blood-films, stained by Gram's method and with methylene blue, showed no micro-organisms; but a drop of blood obtained from the finger with the strictest aseptic precautions, when inoculated on to Agar-Agar with a sterilised platinum needle, gave a pure cultivation of the Streptococcus pyogenes albus. During the next few days the condition remained much the same; some scattered crepitations were heard over the base of the left lung, the murmur over the pulmonary area was louder, and also heard over the aortic orifice with a loud second sound; the murmur was badly conducted upwards; the first sound at the apex was very loud and rough; marked pulsation was felt in the second and third spaces to the left of the sternum.

On November 4th the patient had another rigor, and a second tube inoculated from the blood with the same precautions, again gave a pure culture of S. pyogenes albus. On November 6th treatment by injection of antistreptococcic serum from the British Institute of Preventive Medicine was begun: five injections of 10 c.c. each were administered before November 10th, without any noticeable effect on the temperature, which was taken hourly. The injections were made into the flanks and gluteal regions, and gave rise to considerable pain; probably owing to this cause, the pulse was somewhat—about ten beats per minute—accelerated after them. Between November 6th and 18th, fourteen injections of serum were given: on the 10th, 16th and 17th, no injections were administered, on account of the patient complaining so much of the pain caused by them; on the other days 10 c.c. were given morning and evening. On three or four occasions the temperature fell two or three degrees after an injection, on the others no effect on the temperature could be traced, and on account of the great irregularity of the temperature during the whole course of the illness, it is not probable that the fall of temperature when it occurred had any causal connection with the injection. The sites of injection were the seat of a vivid erythematous blush for some distance around the puncture, which lasted for from a few hours to a day or two, and in several instances of swelling and hardness in the subcutaneous tissues, which were intensely tender to the touch. No effect, other than the acceleration of pulse above-mentioned, and probably due to pain caused by the injection, were noticed on the pulse, respiration, or condition of the urine. The injections gave rise to great pain, making the patient very uncomfortable for hours afterwards, and on this account on November 19th he refused to submit to any more of them. On November 12th, a loud systolic murmur appeared at the apex, and after persisting for two or three weeks, died away again. About this time he complained one day of great pain in the splenic region, and the spleen was found to be further enlarged. On November 14th the patient for a short time appeared much brighter and better, but by the 16th was again as bad as ever.

A few days after the serum treatment was discontinued the blood was again examined for micro-organisms, but this time without any result. The progress of the disease continued unchecked throughout, the patient wasted rapidly and became steadily weaker; he suffered no further pain. Physical examination detected some deficient resonance, and scattered crepitant râles over the bases of the lungs posteriorly. No further signs were heard at the apices of the lungs. The pulmonary murmur remained loud and the spleen enlarged. The abdomen was somewhat distended. There was no albuminuria. On November 18th he brought up a little blood-stained mucus, which contained no tubercle bacilli.

On November 26th, without any improvement in the general condition, which grew steadily worse, the temperature fell and maintained a lower range, the highest point in the twenty-four hours being 100° instead of 102°–103°, until the day of his death, when it rose to 103.° Quiet delirium came on, with some drowsiness, together with increasing weakness; there was tremor of the limbs, with constant plucking at the bedclothes. He gradually sank and died on December 3rd.

P.M.E.—The heart weighed 9 oz.; its cavities were dilated, and the muscle flabby, but looked healthy: there was no endocarditis, the valve segments were normal, and the pulmonary and aortic valves held water. The pericardium was normal. The lungs, liver, spleen, kidneys, pleuræ, and mesentery were found to be thickly studded with innumerable grey miliary tubercles. The lungs were everywhere crepitant, their bases congested, and in the upper lobe of the left lung were a few very small patches of broncho-pneumonia. Over the base of the brain, the bulb and cerebellum, there was an exudation of lymph, in which miliary tubercles were seen. There were no old tubercular lesions or masses in the lungs or other organs, and no caseated glands were anywhere discovered. Scattered follicular ulcers were found in the lower part of the small intestine, which was presumably the source of infection by the bacillus tuberculosis. On microscopical examination of sections of the lungs, spleen, and kidneys, stained by gram's method and by methylene blue, no streptococci or staphylococci were found.

The S. pyogenes albus was found in a pure culture in the blood on two occasions, every precaution being taken to ensure sterilisation of the finger, &c.; attempts were also made to obtain a cultivation of the tubercle bacillus from the blood, but without success. I am indebted to Mr. F. W. Stoddart for his kindness in making the cultivations. The streptococcus was found to have disappeared from the blood after the treatment by the antistreptococcic serum. The local lesions produced by the injections have been described above; no abscesses were produced: an addition of liq. morphinæ ♏v. to the injection was made on two or three occasions, and appeared to mitigate the pain to some extent.

Clinically the diagnosis between ulcerative endocarditis and acute miliary tuberculosis was difficult. After hesitating for a time, the discovery of streptococcus in the blood made me incline to the former diagnosis. In favour of ulcerative endocarditis, in addition to the presence of the streptococcus, were the absence of any family tendency to tuberculosis or of previous tubercular affection in the patient, the shortly antecedent attack of enteric fever, the appearance and disappearance during the course of the illness of cardiac murmurs, and the fact that a very loud rough murmur, unlike a hæmic murmur in character, was heard over the pulmonary area, the occurrence of rigors, the sudden attack of pain over the enlarged spleen suggesting an infarct in this organ, whilst the chief signs of lung affection might from their position be attributed to hypostatic congestion.

---

In the discussion which followed the report of this case Dr. Theodore Fisher referred to the well-known fact that streptococcal infection of the lungs frequently accompanied tubercular disease, and mentioned also that it was not so very uncommon for the heart to be also attacked. He had seen a case in the Bristol General Hospital in which during life there was some doubt as to whether the patient was suffering from malignant endocarditis or from acute tubercular disease of the lungs. At the *post-mortem* examination both diseases were found to be present. Dr. Shingleton Smith alluded to a case of pemphigus foliaceus with continuous pyrexia and indications of a general septic condition, in which antistreptococcic serum had been used; the result was so far undecided, but in spite of much general suppurative cellulitis the pyrexia was now abating, and the condition of the skin had much improved.—Dr. Watson Williams said that in one case of his the temperature, which had been ranging high, rose to 107° after an injection, and if the rise could be rightly attributed to the serum, it would make one chary in some cases.—Mr. C. A. Morton said that he had employed antistreptococcic serum in one case of pyæmia, and although it did not bring down the temperature, yet no rise occurred after its injection.

## Figures and Tables

**Figure f1:**